# Our New York Letter

**Published:** 1887-06

**Authors:** 


### Correspondence.

### OUR NEW YORK LETTER.

*To the Editors of the Atlanta Medical and Surgical Journal:*

The ambulance service has long been an object of pride to New York, for its equal is not to be found in any other city. Perfect as it has been considered, a number of telling improvements in the system have been made within a recent date. At present there are nine hospitals here having an ambulance service, and at each institution two or more ambulances are kept ready for use. At Bellevue Hospital no less than six are employed, as this building is maintained by the city, and their district includes a large portion of New York. The vehicles now used are practically all built on the same plan and consist of four-wheeled rather low-covered wagons with a driver's seat and a bell in front, and a portable seat for the surgeon behind over the tail-board. Only one horse is driven as a rule, but when the streets are heavy with snow two are sometimes used. The ringing of the bell and the word ambulance in large letters secure for this conveyance the right-of-way in any of our crowded streets. Each ambulance has a rubber-covered mattress placed in the bottom, which can be pulled out on small rollers and one end lowered to the ground to receive the patient. An adjustable stretcher with blankets and pillow lies on the mattress. The other equipments include a medicine-chest, splints, surgical dressings and bandages. Besides these, other articles are sometimes carried, as a strait jacket, stomach-pump and a hand-lantern. The medicine-chest is usually well supplied with drugs commonly used in emergencies, but the ambulance surgeon rarely uses them. Whisky is about the only medicine he gives his patients to any extent, for he meets with many cases of shock and collapse from severe accident, where heart stimulation is the primary indication for treatment. Emetics are frequently administered in poisoning cases, but the surgeon's motto is generally to have the patient driven to the hospital as quickly as possible, where treatment may be resorted to in a much more satisfactory manner than on the street. At some of the hospitals an attempt is made to carry out the antiseptic treatment of wounds in their ambulance cases, and for this purpose solutions of corrosive sublimate and carbolic acid are used, after which iodoform gauze dressings are applied. The position of ambulance surgeon is generally filled by the surgical junior or senior members of the hospital house staff. His duties at first are apt to be accompanied with fear and trembling, as on every call there is an uncertainty as to what he may encounter. But he soon learns that it is only necessary to resort to temporary measures in the majority of his cases, and the temerity gives way to a feeling of self-confidence in which he thinks himself capable to cope with anything, after which the ambulance service possesses a peculiar fascination to him. In a crowd the surgeon may be recognized by his cap bearing the name of his position in gilt letters, and at some hospitals a neat blue uniform with brass buttons is worn. He also carries a small hand-satchel containing roller bandages. The horses employed are fast and strong, and a drop-harness, similar to that used in the fire department, enables the ambulance to turn out in a few seconds. There are several ways of sending out a call in case of accident or sickness. The most common is by telegraph or telephone through the police wires. An accident on the street being reported to a policeman, he goes to the nearest police station and has the sergeant here telegraph to the police central office. At this place an operator sends the message to the hospital nearest the scene of accident. Some of the police stations, however, have direct communication with the nearest hospital. An ambulance may also be called by the fire telegraph, either from the automatic boxes on the street or from a fire engine-house. Only chiefs of the fire department are allowed to send out calls, and this system is employed for accidents at fires, or near engine-houses, and in great calamities where all the ambulances are wanted. When a call is sent over the fire wires the same signal rings simultaneously in all these hospitals and the institution nearest the accident is expected to respond. The existing rivalry, however, commonly leads to two or more ambulances from different hospitals responding, and the result is a very lively race, for the first surgeon on the ground claims the patient. This rivalry is a very good thing for the patient, as it secures the quickest possible attention if nothing else. These races always furnish excitable enjoyment for doctor and driver, who pride themselves on their horses and boastfully talk of their successful trips. In one precinct there are five different police alarm systems being experimented in at present. Only one, however, is in use and consists of automatic telegraph boxes on the lamp-posts, which enable the police to communicate directly with the hospitals. Calls may now also be sent from the elevated railroad stations. By an arranged set of signals different calls may be sent out as a "hurry up" or a call for one, three or all the ambulances in the city. This latter signal is sent out on an average of twice a year. It would thus seem an easy matter to obtain an ambulance when necessary, and yet there sometimes appears to be considerable red tape about this procedure.

A doctor recently was called to see a man who had just broken his leg. The patient was unable to pay for the necessary attention, and decided to go to a hospital, so the doctor volunteered to have an ambulance sent immediately. He first went to an engine-house one block distant, but was told by the captain that it was contrary to the department's rules to telegraph for an ambulance when the patient was in his home. Then he went to an elevated road station, but was informed that such a request must be made by a policeman. He started to find one of these officials, but it was the time of night when policemen are scarce and the doctor finally gave up in disgust.

Surprisingly large sums of money are paid by some of our residents for medical services. One lady, worth $10,000,000, who died here recently, paid her medical adviser $6,000 a year. The widow of one of our richest merchants was in the habit of consulting three physicians at an expenditure of $40,000 yearly. When the doctors disagreed she abided by the decision of two of them. Another wealthy lady pays her physician an average of $20,000 a year, her checks always exceeding the amount of the bill. By over-remuneration she imagines that the doctor will render her better service. $10,000 a year for medical advice is paid by one individual who is said to enjoy the best of health.

The Academy of Medicine intends erecting a new building and a committee is at present in search of a suitable location. For the house and property $250,000 will be expended. Many large bequests have been made to the academy and the organization has grown wealthy. Originally its meetings were held in Wooster street, and from here it removed to the present quarters in West 31st street. The entire four floors are occupied, there being a library of 35,000 books and about 200 medical journals on file. A. F.

*New York, May, 1887.*

